# What Does a Language-And-Vision Transformer See: The Impact of Semantic Information on Visual Representations

**DOI:** 10.3389/frai.2021.767971

**Published:** 2021-12-03

**Authors:** Nikolai Ilinykh, Simon Dobnik

**Affiliations:** The Department of Philosophy, Linguistics and Theory of Science (FLoV), The Centre for Linguistic Theory and Studies in Probability (CLASP), University of Gothenburg, Gothenburg, Sweden

**Keywords:** language-and-vision, multi-modality, transformer, representation learning, effect of language on vision, self-attention, information fusion, natural language processing

## Abstract

Neural networks have proven to be very successful in automatically capturing the composition of language and different structures across a range of multi-modal tasks. Thus, an important question to investigate is how neural networks learn and organise such structures. Numerous studies have examined the knowledge captured by language models (LSTMs, transformers) and vision architectures (CNNs, vision transformers) for respective uni-modal tasks. However, very few have explored what structures are acquired by multi-modal transformers where linguistic and visual features are combined. It is critical to understand the representations learned by each modality, their respective interplay, and the task’s effect on these representations in large-scale architectures. In this paper, we take a multi-modal transformer trained for image captioning and examine the structure of the self-attention patterns extracted from the visual stream. Our results indicate that the information about different relations between objects in the visual stream is hierarchical and varies from local to a global object-level understanding of the image. In particular, while visual representations in the first layers encode the knowledge of relations between semantically similar object detections, often constituting neighbouring objects, deeper layers expand their attention across more distant objects and learn global relations between them. We also show that globally attended objects in deeper layers can be linked with entities described in image descriptions, indicating a critical finding - the indirect effect of language on visual representations. In addition, we highlight how object-based input representations affect the structure of learned visual knowledge and guide the model towards more accurate image descriptions. A parallel question that we investigate is whether the insights from cognitive science echo the structure of representations that the current neural architecture learns. The proposed analysis of the inner workings of multi-modal transformers can be used to better understand and improve on such problems as pre-training of large-scale multi-modal architectures, multi-modal information fusion and probing of attention weights. In general, we contribute to the explainable multi-modal natural language processing and currently shallow understanding of how the input representations and the structure of the multi-modal transformer affect visual representations.

## 1 Introduction

The ability of transformers to capture contextualised representations and encode long-term relations has led to their successful application in various NLP tasks (Vaswani et al., 2017; Devlin et al., 2019; Radford et al., 2019). Their large size, layer depth and numerous multi-head self-attention mechanisms are the main reasons for their excellent performance. However, the structure of such ever-larger models imposes new challenges on understanding and explaining their inner workings. Since there is no clear cognitive motivation behind tremendously successful transformers (Rogers et al., 2020), a set of sophisticated methods is required to examine how information is processed and what is learned by such models. Multiple explainability methods and tools have been proposed in the ‘BERTology’ field, which investigates whether transformers can learn helpful information. In these approaches, self-attention is typically inspected for the presence of specific *linguistic knowledge* as a product of cognition. For example, some research has focused on identifying valuable information for syntactic, co-referential, and translation tasks (Raganato and Tiedemann, 2018; Belinkov and Glass, 2019). Notably, Vig and Belinkov (2019) show that more complex linguistic phenomena are captured in deeper attention heads of the model, building on top of much simpler knowledge present in earlier layers of the model. Such hierarchical learning of linguistic information is further exemplified by showing that proper nouns are learned in deeper layers, and low-level constructs such as determiners are captured in lower layers. Others have inspected each attention head individually for the specific type of information (Voita et al., 2019), or even tried to explain attention by comparing it to human input in particular contexts (Hoover et al., 2020). However, it has been emphasized that attention is not always an explanation of the linguistic knowledge learned by the model (Jain and Wallace, 2019), and several other factors have to be taken into account when explaining such models (Kobayashi et al., 2020). Some other popular explainability methods include neuron-based analysis and transfer learning (Rethmeier et al., 2020) and promising gradient-based analysis, which directly reflects the knowledge learned by the model (Wallace et al., 2019). However, it has been recently shown that it is relatively easy to manipulate and corrupt gradient-based explainability methods (Wang et al., 2020).

The transformers have also taken by storm the field of computer vision, one of the last bulwarks of CNNs. Dosovitskiy et al. (2021) have shown that vanilla transformer demonstrates impressive results on the task of image classification if supplied with simple BERT-style image representations (e.g., 2D image patches). Interestingly, the authors show that the vision transformer can gradually increase its attention on the semantically plausible parts of the image, structuring its visual knowledge. Specifically, attention heads in surface layers uniformly attend to many different areas in the image, with attended patches relatively close to each other. In contrast, deeper attention heads focus on specific image patches, while the distance between attended patches becomes larger. Caron et al. (2021) observed that attention heads in vision transformer capture class-specific features of objects (e.g., shapes, parts), which can be indicators of emerging *visual knowledge* of the world. Interestingly, the authors show that the model focuses more on “class-specific” features when trained with self-supervision. In contrast, using object labels in a standard supervised setting dissolves its focus and re-distributes the model’s attention across different parts of the image. This finding raises a question of the effect that language has on visual representations^1^. While more focus on a single object might be beneficial for image classification, a more sophisticated multi-modal task (e.g., image captioning) requires scene-level knowledge about objects and relations between them. Thus, more global attention shaped by the conceptual knowledge from language is required for such tasks. One shortcoming of many current vision-only transformers is that the representations learned by such models lack grounding in the broader relational knowledge between different objects expressed in image descriptions. This characteristic of the vision transformers provides additional motivation for the current study and our exploration of how language-and-vision transformers can benefit from a combination of two modalities.

Somewhat surprisingly, only recently multi-modal transformer representations have started getting attention from scientists. Cao et al. (2020) probe the pre-trained multi-modal transformers for several language-and-vision tasks and show that these models encode a variety of useful textual, visual or cross-modal representations. However, a better understanding of how multi-modal representations are structured and implicitly learned is currently missing in the literature. Additionally, we need to know what is the role of explicit factors, such as the way the image is fed to the model. Therefore, in this paper, we address the problem of transparency of multi-modal representations and experiment with the two-stream image captioning transformer introduced by Herdade et al. (2019). In this transformer, each modality (language and vision) is first attended separately by modality-dependent self-attention, and then the two are fused by the third component, cross-modal attention. The separation of the system into three modules allows us to examine fine-grained uni-modal representations in the multi-modal architecture. A dedicated module for merging of visual and linguistic information allows us to study how they are fused. Thus, a two-stream transformer can utilise the combination of conceptual knowledge of how objects can be distributed and related to each other based both on linguistic and visual information. Such models learn to perform a variety of tasks:

⋄ Visually parse the scene: find patterns/invariances that are visually salient across different visual contexts (vision stream).
⋄ Extract knowledge from linguistic descriptions: find salient patterns between word representations and sequences of words (language stream).
⋄ Combine both information types to make visually and linguistically dependent representations which are grounded in how we structure and label the world as reflected in language and what we observe visually (cross-modal stream).

The analysis of representations learned by the multi-modal architectures is also relevant in the context of the call for a change in what semantic representations we use in natural language processing (Bender and Koller, 2020; Bisk et al., 2020). As the authors point out, semantic representations learned from word embeddings are insufficient and grounded representations are required. Investigating multi-modal models allows us to study how such representations differ from representations learned in completely uni-modal architectures. In Ilinykh and Dobnik (2021), we demonstrate that visual knowledge indirectly affects language representations in the multi-modal transformer. Our experiments show that the self-attention in the language stream becomes more focused on previously generated nouns, aligning with visual modality and image objects. A natural continuation of this analysis is to examine whether the structure of visual representations is influenced by conceptual knowledge of the world, reflected in language. Therefore, we examine how a multi-modal transformer proposed by Herdade et al. (2019) organises and structures learned knowledge of the visual modality. We focus on the vision stream and inspect 1) how visual knowledge is represented in a transformer as exemplified by self-attention, 2) how visual knowledge is affected by the overall training task, which is image caption generation, and 3) whether the observed attentional patterns are intuitively interpretable to us.

We address the following questions:

1) Given the multi-layered nature of transformer blocks and, therefore, differences in input representations at each step, self-attention heads at each layer are expected to differ in the type of knowledge that they encode. We investigate what kind of knowledge is captured by different layers by examining visual self-attention patterns between objects.
2) Knowing that both language and perception have a hierarchical structure (Tenenbaum et al., 2011), we also expect hierarchical learning of visual information in transformers. Is there a progression of attended representations from low-level local relations to high-level global dependencies between objects corresponding to our conceptual knowledge? Moreover, is there a connection between learned dependencies and the input representations, which can be either semantically informed (e.g., object detections) or disentangled from any conceptual meaning (e.g., image patches)?
3) Does the language task have an effect on visual representations? Due to the back-propagation mechanism and the multi-modal fusion module, representations of one modality might contain artefacts of another modality in the two-stream multi-modal transformer (Ilinykh and Dobnik, 2021). Is conceptual linguistic knowledge *implicitly* reflected in visual self-attention?

The remainder of the paper is organised as follows. In Section 2.1, we review the model’s architecture and introduce the notion of the *attention link*, an important concept that we use to interpret knowledge captured by the model. We also describe our experimental setup. Then, we provide a short analysis of how the input representations we use in our experiments might affect what and how the model learns (Section 2.2). We proceed to the main experiments in Section 3. Section 3.1 and 3.2 describe the analysis of the knowledge in all layers in terms of thematic relatedness of the objects, visual proximity and strength of the attention links. In Section 3.3, we identify the knowledge split in what is learned by earlier and deeper layers of the model by analysing representations from different layers for similarity with each other. We also examine the spread of attention between objects as shown by attention patterns in the model’s layers. Section 3.4 describes our analysis of whether the high-level knowledge from language modality can be detected in layers of visual self-attention in some form. We inspect representations learned with a different input type (e.g., image patches) in Section 3.5 and compare them to the knowledge captured when using object detections. Finally, in Section 4, we connect our results with studies on human cognition. We conclude with a summary of how our experiments contribute to a better understanding of large-scale neural models and identify possible research questions for future work (Section 5).

## 2 Materials and Methods

### 2.1 Two-Stream Multi-Modal Transformer

Traditional multi-modal architectures (Xu et al., 2016; Lu et al., 2017) learn a single set of attention weights with RNN or LSTM attending over convolutional features. In comparison, current transformer-based architectures encode information with multiple attentions, either processing each modality independently as in two-stream or multi-stream models (Lu et al., 2019; Tan and Bansal, 2019) or simultaneously (one-stream) (Chen et al., 2020; Su et al., 2020). The separation of different modalities (*multi-stream*) allows us to 1) inspect the fine-grained attention patterns learned at each modality or level of organisation and 2) examine the effect of information fusion on representations of each modality. Therefore, we use the image captioning transformer by Herdade et al. (2019), which is based on the standard transformer model (Vaswani et al., 2017), consisting of three different self-attention blocks. Effectively, the modular design allows two-stream architectures to learn to encode contrasting requirements of each modality, similar to the conditions imposed by human vision and human language. In addition, the third module (cross-modal attention) fuses both types of information which is intuitively comparable with how humans combine perceptual and conceptual understanding to describe the world.

We focus on image captioning because this generation task represents a basic linguistic pragmatic case. In comparison, the VQA task (Antol et al., 2015) is a multi-label classification problem, which requires more focus on specific objects mentioned in a question. Visual dialogue (Das et al., 2017) imposes additional challenges, which often require knowledge beyond images and texts, e.g. memory and tracking of attention focus on objects. We believe that inspecting multi-modal representations for visual dialogue is out of the scope of the current paper and hope to address this problem in future work.

The primary difference between the model introduced by Herdade et al. (2019) and other two-stream architectures (notably, LXMERT (Tan and Bansal, 2019) and ViLBERT (Lu et al., 2019)) is the method that is used to encode spatial information about objects. Both LXMERT and ViLBERT do not incorporate any object relative geometry. Instead, they simply utilise coordinates of bounding boxes or their spatial location. In addition, we find BERT-inspired LXMERT and VilBERT to be more suited for learning general multi-modal representations, while the multi-modal transformer by Herdade et al. (2019) is particularly tailored for better use of modalities for a specific task of caption generation.

**FIGURE 1:**
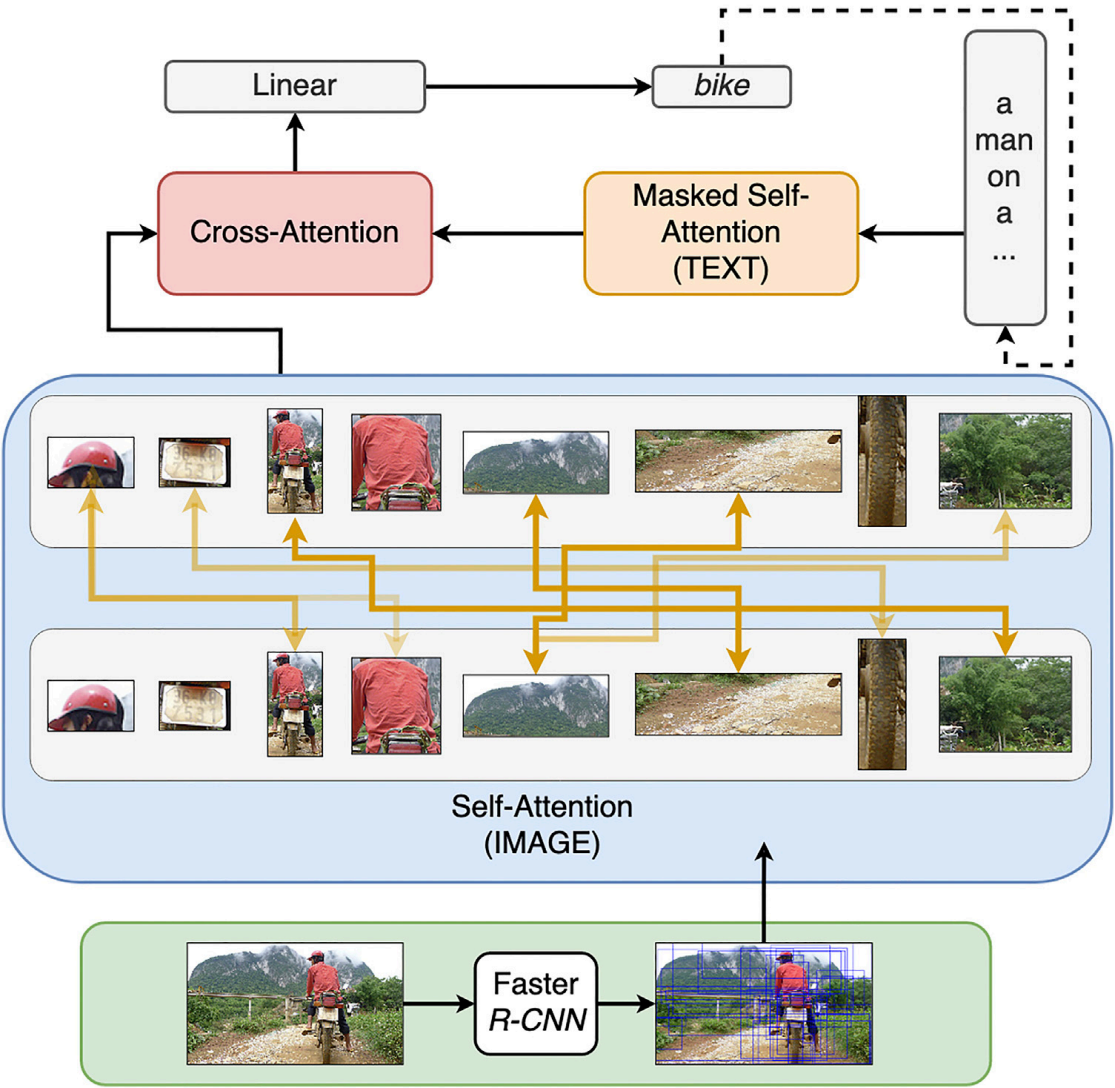
The architecture of the multi-modal transformer and a detailed visualisation of its image encoder (visual self-attention). The model consists of three parts: self-attention on visual information, masked self-attention on textual input and cross-attention, which learns multi-modal fusion. The predicted word is concatenated with the previously generated words and passed as the new input to the masked self-attention. The image encoder takes (i) visual representations of objects produced by a pre-trained faster R-CNN and (ii) geometric representations between detected objects. The self-attention operates at the level of detected image objects, building attention links of various strength between them. The intensity of the orange lines indicates the intensity of the attention links between objects. The attention links are created by every attention head in all layers of the image encoder and, if present, can vary in terms of attention strength.

Figure 1 describes the main components of our model. The model consists of three modules, where each of them operates with different input representations and consists of *L* = 6 layers and *H* = 8 attention heads in each of these layers. For the masked self-attention (the orange box), we mask all words (*w*_*t*+1_, … , *w*_*T*_), which follow the word at the current timestep *w*_*t*_, with the [*MASK*] token. We set *T* to 16. Such design is necessary for the uni-directional task of image caption generation in which sequences of words are formed gradually as the description unfolds. The masked self-attention produces representations for the next word *w*_*t*+1_ given the previous context and the most recently generated word (*w*_1_, … , *w*_*t*_). In our experiments, we refer to the masked self-attention module as the *text encoder* since this part of the model effectively learns the representation of the next word from the text. The cross-attention module later uses this representation with the output from visual self-attention to predict the next word along the lines of the standard decoding process.

Self-attention on visual information (the blue box) is another important part of the model. In a typical text-based transformer such as text-to-text transfer transformer (T5) (Raffel et al., 2020), this part of the model learns textual representations. However, in a multi-modal task, the self-attention is performed on image objects, which are detected and labelled prior to the model’s training. We refer to this part of the model as *image encoder* (the motivation behind the naming is similar to text encoder). To prepare input for the image encoder, we use the released feature extractor (Anderson et al., 2018)^2^ that has been pre-trained on object annotations from Visual Genome (Krishna et al., 2016). This model is based on Faster-RCNN (Ren et al., 2015) with the ResNet-101 (He et al., 2016) as its visual backbone. Its output consists of visual and geometric features of objects within bounding boxes, labels (“cup”) and attributes (“red”). Each attribute-label pair is accompanied by a specific score, indicating the model’s confidence about the correctness of the attribute. In our experiments, we keep only such attributes that have a confidence score of 0.1 or more. Each detected object is represented by a single feature vector $f_{n}\in R^{1\times D}$ , where *D* = 2048. All object features form a feature set **F** = {**f**_1_, … , **f**_*N*_} with *N* = 36.

In addition, Herdade et al. (2019) also extract geometric features **G** = ⟨*x*, *y*, *w*, *h*⟩ which represent the centre coordinates, width and height of every object in the image. The image encoder is provided with geometric representations, used as the positional encoding of object representations. The idea of using positional information in visual self-attention is motivated by the fact that objects in images do not have a natural order in terms of their arrangement, unlike words, and supplying models with such geometric knowledge might provide information about the topology of objects. Here we briefly describe how visual and geometric features are combined, while referring the reader to Herdade et al. (2019) for more information. First, inspired by Hu et al. (2018), a 4-dimensional displacement vector between every two objects is computed. Similar to Vaswani et al. (2017), authors learn positional (geometric) embeddings by applying the sinusoidal function to the displacement vector and get a high-dimensional intermediate geometric representation $E\in R^{1\times N\times N\times d}$ . This representation is then flattened and multiplied with a learned linear matrix $W_{g}\in R^{1\times H\times N\times N}$ and passed through ReLU non-linearity to obtain *geometric attention weights* as follows:

$$\Omega^{G}=EW_{g}.$$ (1)

At the same time, visual representations are used to learn queries **Q** and keys **K**, two standard parameters of the self-attention:

$$U=Dropout\left(ReLU\left(W_{p}F\right)\right),\;Q=UW_{q},\;K=UW_{k},$$ (2)

where **F** is the set of visual features or output of the previous layer, $W_{p}\in R^{D\times M}$ , $W_{q}\in R^{1\times H\times d\times N}$ and $W_{k}\in R^{1\times H\times d\times N}$ are learned during training. **W**_*p*_ is used to reduce the dimension of visual features resulting in **U**. The *visual attention weights* are calculated as follows:

$$\Omega^{V}=\frac{QK^{T}}{\sqrt{d_{k}}}.$$ (3)

$1/\sqrt{d_{k}}$ is a scaling factor, which provides efficient learning for larger inputs since their size can affect the gradient of the softmax function and make it too small. Next, geometric **Ω** ^*G*^ and visual **Ω** ^*V*^ attention weights are combined as follows:

$$\Omega =\log\left(\Omega^{G}\right)+\Omega^{V},$$ (4)

where   log is used to normalise the distribution of geometric weights. Finally, the third standard parameter of transformer’s self-attention, value **V**, is multiplied with the combined features:

$$V=UW_{v},\;{head}_{h,\ell }\left(F\right)=softmax\left(\Omega \right)V,$$ (5)

where $W_{v}\in R^{1\times H\times d\times N}$ is the learned matrix, Ω is the *N* × *N* matrix with combined attention weights, *head*_*h*,*ℓ*_ corresponds to the specific attention head *h* in the layer *ℓ*. The authors set *M* to 512, *d* to 64 and *d*_*k*_ to 64. Note that the geometric features are provided to every visual self-attention layer. Every attention head at each layer learns its own set of transformer parameters (**Q**, **K**, **V**) and merges *the fixed set of geometric features with the output from the previous layer*, using it to produce the final result. In the end, the output of the last layer is passed to the cross-attention module (the red box), which attends to both visual and textual representations to generate the next word *w*_*t*+1_.

In our experiments, we are using attention weights between objects predicted by individual attention heads *h* within each layer *ℓ* of the image encoder. We refer to them as attention links to emphasise their role in connecting different objects. The attention weights are extracted from the image encoder according to Eq. (3). Then, we apply softmax over these representations to obtain the set of attention links. The notion of attention link represents observable attention between two identical or different objects. The attention link is *strong* if the weight that it establishes between the objects is close to 1. Otherwise, the attention link is *weak* if it is close to 0 which indicates no attention. Note that the attention weights of a specific head will typically focus on particular objects rather than all objects.

In terms of the dataset, we use the Karpathy test split (Karpathy and Fei-Fei, 2015) of the MSCOCO image captioning dataset (Lin et al., 2014) to test our model and extract attention weights. The test split consists of 5,000 images with five captions per image, while train and validation splits contain 113,000 and 5,000 images, respectively. We take the pre-trained checkpoint of the multi-modal transformer released by Herdade et al. (2019) ^3^. The choice of the checkpoint was based on the CIDEr-D score (Vedantam et al., 2015). The released checkpoint is not perfect and does not achieve human performance in generating image descriptions. In this evaluation exercise, we are interested in the attention that a model would predict for natural, therefore, human-generated descriptions. Hence, we perform the model testing in a teacher forcing setting: every next generated word is replaced with the corresponding word from the ground-truth captions and used as part of the following input to the model. We collect attention links from the image encoder for every caption generated this way.

### 2.2 Learning From Object Detections, Not Pixels

The first layer of visual self-attention is provided with visual features of detected objects and geometric information about them. These objects become connected by attention links of various strengths at every layer during learning, resulting in many pairwise relations. We ask whether learned attention links are *influenced* in any way by the type of input that the model uses. In particular, we want to know how the input data guides the model’s learning and what knowledge the model builds between attended objects.

**FIGURE 2:**
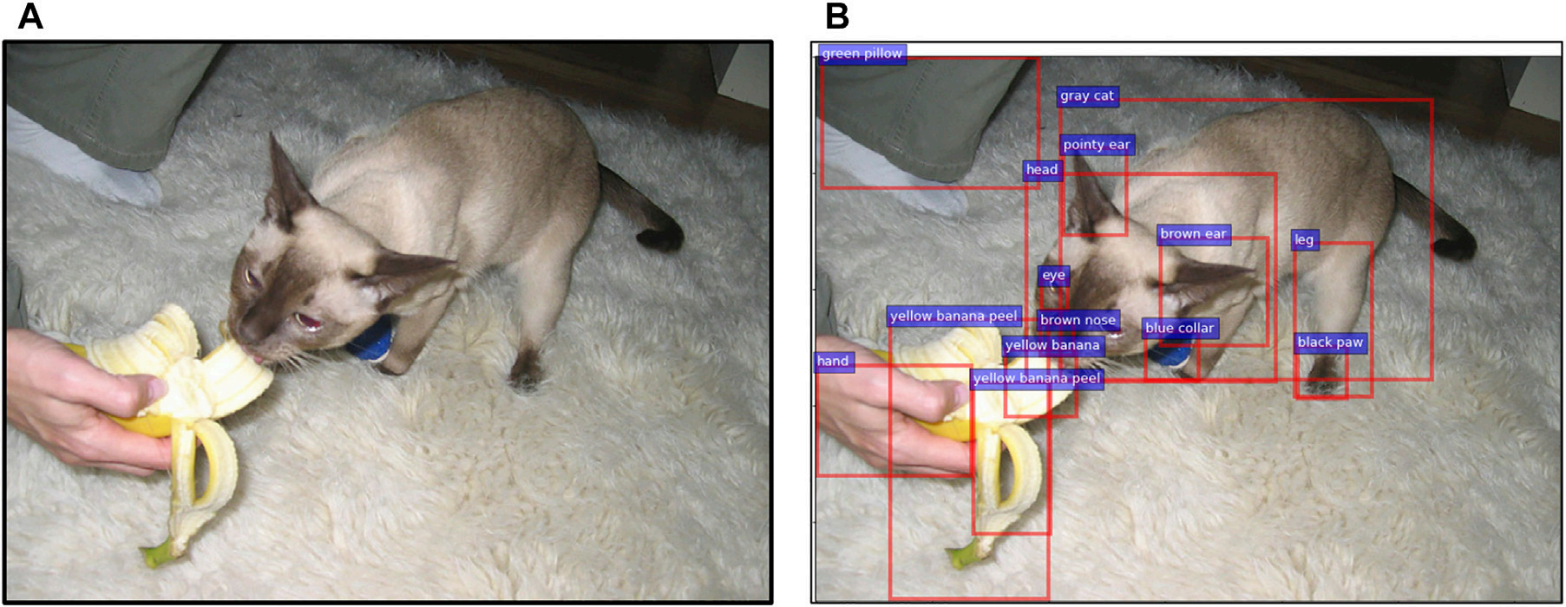
Example of object detection output from the MSCOCO dataset. The original image **(A)** and a subset of the detected objects represented by bounding boxes, labels and attributes **(B)**. The caption for the original image: “a cat that is eating some kind of banana”.

Figure 2 shows an example of the output from the object detector. The sub-figure B demonstrates that many bounding boxes capture parts of what we would consider being the same entity: e.g. “black paw” of the “leg” which belongs to the “cat” object. The extractor can detect the same object multiple times (“yellow banana peel”), and identifying the wrong object is also possible. Therefore, the input to the first layer of the transformer consists of features of objects of different granularity, ranging from entire entities (“cat”) to their parts (‘head’). In comparison, vision transformers (Dosovitskiy et al., 2021) start with the analysis of images on the pixel level when only perceptual information is available. The image encoder in our model is expected to benefit from the *conceptual knowledge of the objects*, as provided by the object extractor, pre-trained on *human* annotations of visual scenes. The model thus might be primed to group various objects into larger concepts (“ear”, “leg”, “paw” belong to “grey cat”), acquiring lower-level cognitive information about part-whole relations and a better understanding of local relations between objects.

In addition, the input is represented by visual features of bounding boxes representing objects *over the entire image* and their geometric information, and so the transformer is expected to capture a global understanding of an image. The self-attention in transformers can be interpreted as a parallel to larger receptive fields (Parmar et al., 2018) since it operates on a group of objects across the image. In contrast, convolution operators fall back on determining dependencies and relations between objects, while efficiently detecting objects and extracting their features (Kelleher and Dobnik, 2017). For instance, relations such as “the leg is next to the ear” or “the banana is in front of the nose” will not be learned by CNNs because of the small size of the convolutional kernel while objects and their features (“leg”, “ear”, “banana”, “nose”) will be detected (Kelleher and Dobnik, 2017; Ghanimifard and Dobnik, 2018). Thus, one type of knowledge that is encoded by attention weights is the knowledge of long-distance visual dependencies between objects (see, for example, the study by Ghanimifard and Dobnik (2019)).

Self-attention can capture both local and global knowledge as it is not limited by the size of the receptive field nor by the kind of information that we present to it in the vector, i.e. we can mix features of a different kind. The knowledge that we give to the transformer is a higher-level knowledge that is the output of the feature extractor, which detects objects based on the convolution algorithm. In the following experiments, we examine the attention links established by different layers of visual self-attention and the type of relations they resemble by learning from features of detected objects.

## 3 Experiments

### 3.1 Thematic Analysis of Attended Objects

We inspect to what degree objects that are linked by attention are thematically related to each other. The output of the feature extractor provides us with both object features and object descriptions. Thus, we can measure the association between objects through semantic similarity of their descriptions. We represent the descriptions of detected objects as word embeddings by using the Word2Vec model (Mikolov et al., 2013) pre-trained on Google News dataset and available in Gensim (Řehůřek and Sojka, 2010). Next, we cluster the label embeddings into *C* clusters, using the k-means algorithm for clustering (Lloyd, 1982). Prior to clustering, we remove all attributes from the object descriptions since they provide an irrelevant dimension of comparison and might affect the clusters, making them less object-specific. For example, excluding “grey” from “grey table” and “grey cat” prevents situations when two descriptions of thematically unrelated objects (table and cat) are placed in the same semantic cluster of grey objects because of the shared colour dimension. We examine the cluster membership of every pair of detected objects. If they are in the same cluster, then the two objects are thematically associated; otherwise, the two objects are not related thematically. We set the number of thematic clusters *C* = 3 as this is the average number of objects described by humans in noun phrases from captions. This indicates that there are on average three relevant objects present in images.

We calculate the average proportion of attention links between objects that are within the same semantic cluster according to :

$$Prop\left(\alpha |\ell ,h\right)=\frac{1}{\left|IMG\right|}\times \frac{\sum_{img\in IMG}\sum_{i=1}^{N}\sum_{j=1}^{N}\alpha \left(n_{i},n_{j},clust\left(n_{i}\right)=clust\left(n_{j}\right)\right)}{\sum_{img\in IMG}\sum_{i=1}^{N}\sum_{j=1}^{N}\alpha \left(n_{i},n_{j}\right)},$$ (6)

where *ℓ*, *h* stands for a specific layer and attention head, **IMG** is the test set of images, *N* is the number of objects in an image, *clust* denotes the cluster of a specific object, *α* is the attention link between the objects.

**FIGURE 3:**
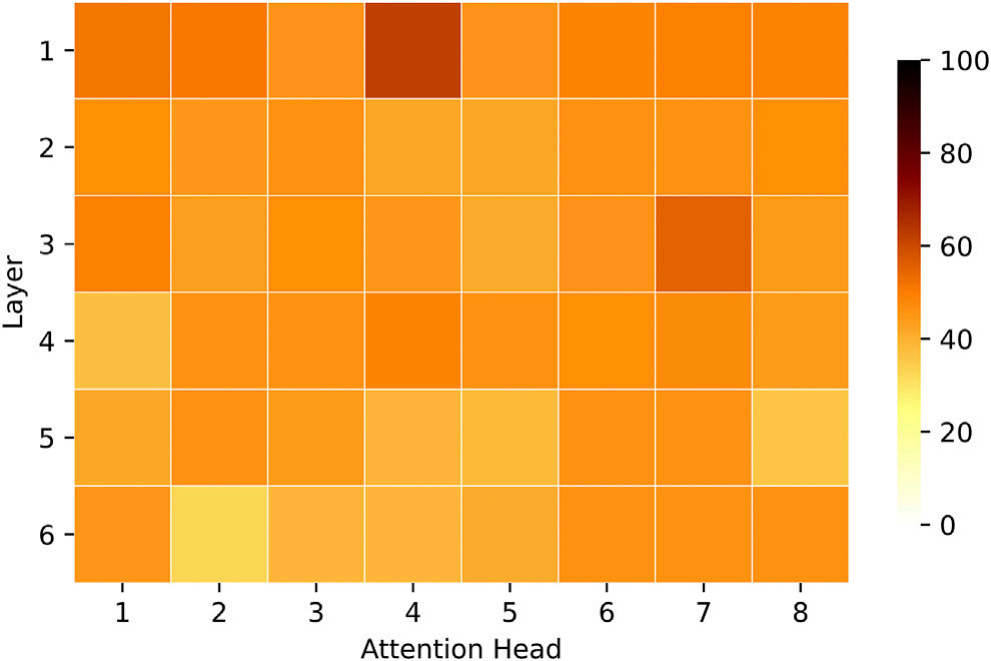
For every attention head, we show the proportion (in %) of attention links between objects in the same semantic cluster vs all attention links disregarding the cluster. The results are averaged across all images in the test set. Attention heads and layers are shown in horizontal and vertical axes accordingly. The darker the colour, the larger the ratio. The colour scale on the right indicates the range of attention proportions (minimum is 0, maximum is 100).

The results in Figure 3 demonstrate that surface layers encode visual properties within thematic categories, whereas deeper layers focus on visual properties that go beyond the automatically identified thematic categories. For example, the attention links in the first layer are created between objects within the same thematic cluster on average in 50% of cases, compared to 41% in the last layer. More specifically, the top-5 (in the descending order) attention heads that link thematically related objects are all located in the first three layers of the visual encoder (1–4, 3–7, 1–1, 1–2, 3–1)^4^ with four of them located in the first layer. The best attention head (1–4) builds links between thematically related objects in 62% of cases, while the head that builds such connections the least does so in 33% of cases (6–2). The results indicate that the knowledge of thematically related objects, which possibly includes local dependencies (e.g., part-whole relations), is primarily captured in the first layers.

**FIGURE 4:**
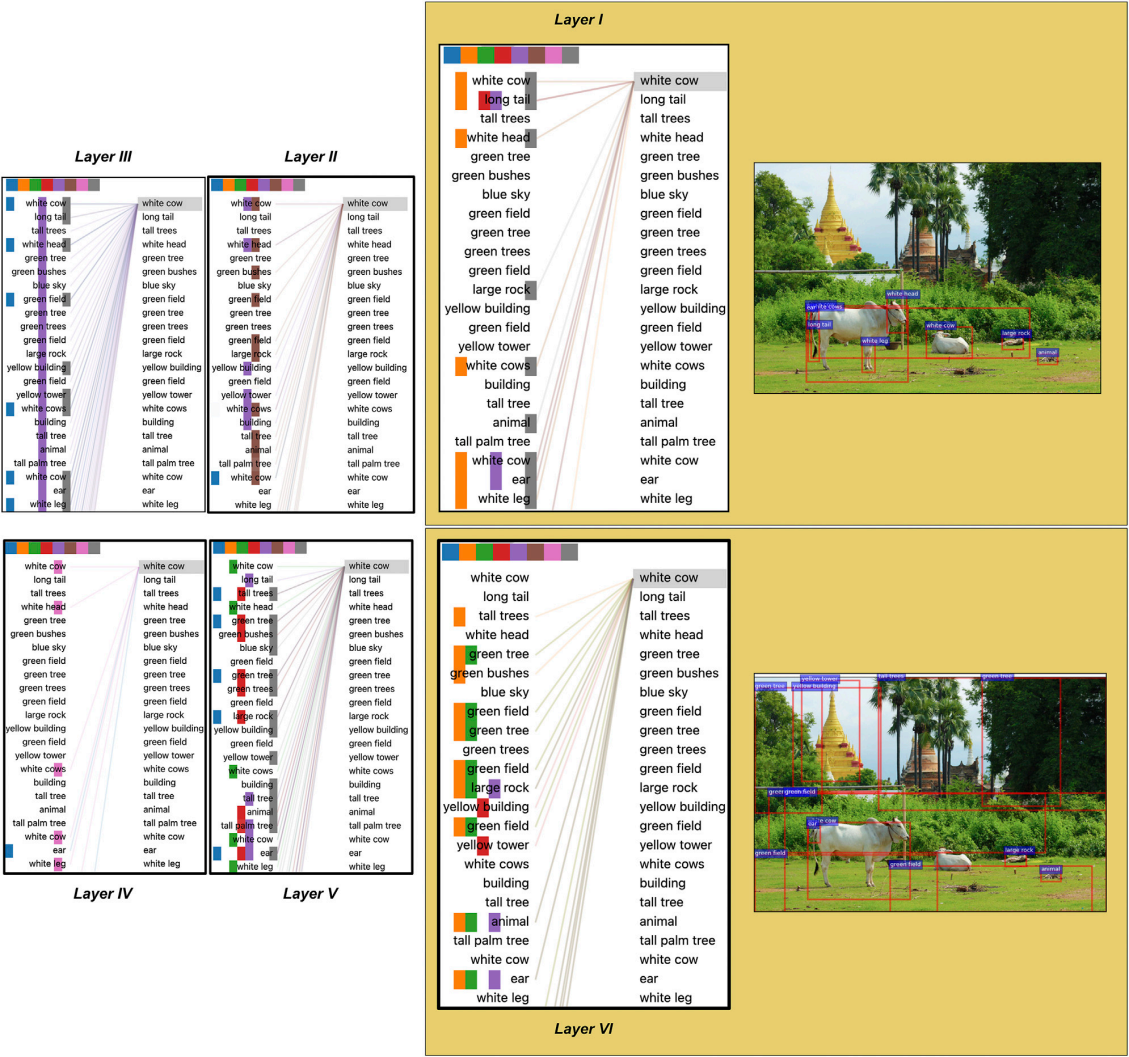
An image with (i) bounding boxes for a subset of detected objects (in red), and with (ii) visual self-attention connections for all six layers between this subset of 36 objects. Caption for the image: “two cows outside, one laying down and the other standing near a building”. Differently coloured squares on top of each layer visualisation indicate different attention heads. Each layer is displayed with two identical lists of objects: the left column shows the source objects, while the right column depicts the target objects which receive attention from source objects.

**FIGURE 5:**
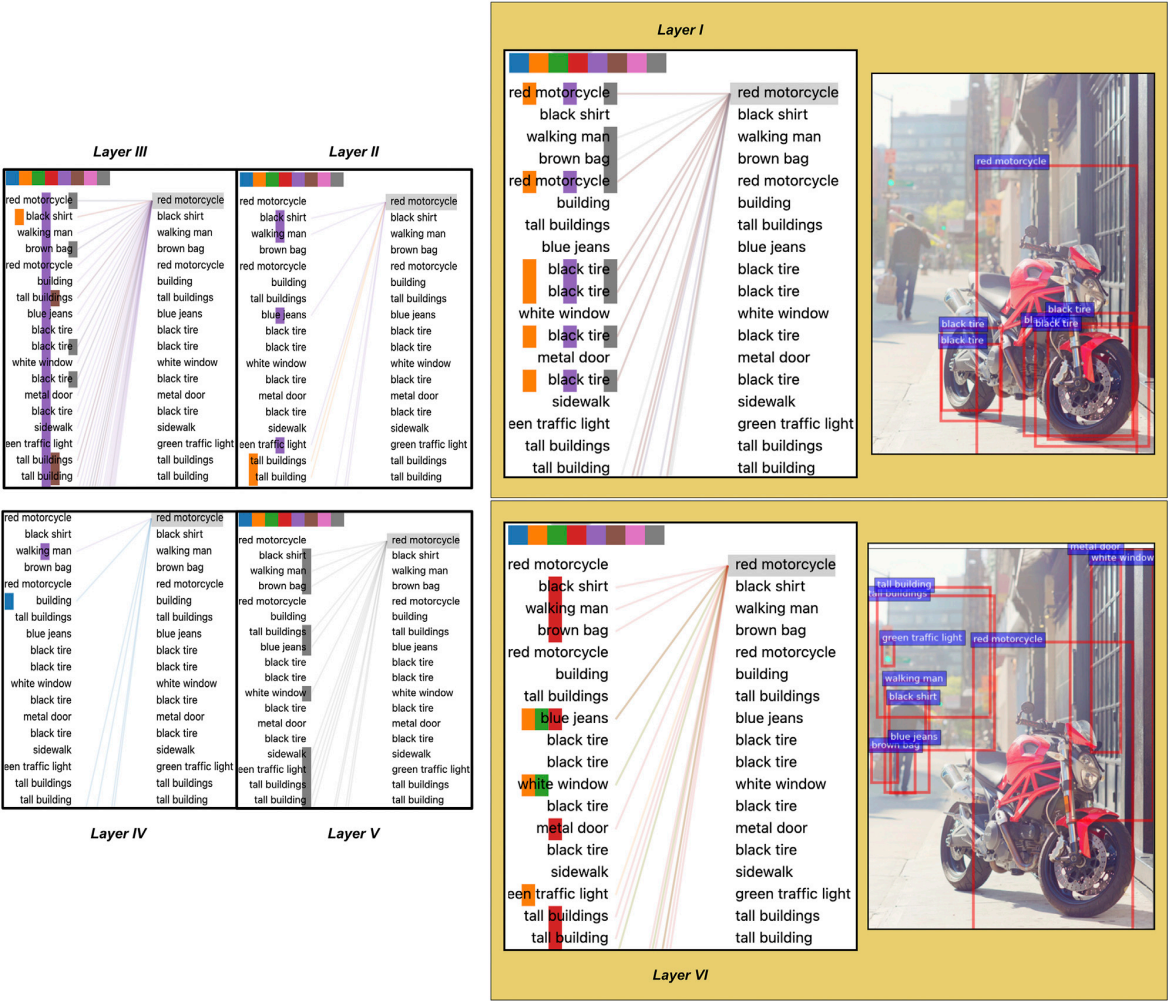
An image with attention links between objects. Caption for the image: “red motorcycle parked outside of large building in the city”. All other details about this figure are as for Figure 4.

We support the results with the visualisations of attention links between objects in different layers in two images in Figure 4 and Figure 5. We use the tools provided by Vig (2019) for visualisations. Looking at Figure 4 (layer 1), we see that “white cow” is strongly attended by objects thematically related to cows as entities, indicating the learning of local dependencies. For example, “long tail”, “white head” and “white leg” are all parts of the entity “white cow”. Similarly, in Figure 5 (layer 1), attention heads relate parts of an object (“tires”) to the object itself (“motorcycle”). However, the attention links in deeper layers capture a different kind of knowledge. In particular, in Figure 4 (layer six), the attention heads link “white cow” with objects describing animal’s surroundings: “tall trees”, “green tree”, “yellow building”. At the same time, in Figure 5, attention heads in layer six also connect “red motorcycle” with other objects in the scene: “walking man”, “metal door”, “green traffic light” etc. These differences in attention between objects in earlier and deeper layers indicate that earlier layers might focus on learning relations, which are more local such as part-whole relations. In comparison, deeper layers capture a different type of thematic relatedness, e.g. relations between *different* objects.

In earlier layers, the model operates *only* with low-level visual features of bounding boxes. Nevertheless, it can capture *semantic* similarities between objects in terms of their labels. This semantic information must come from elsewhere, possibly as a side effect of image segmentation into objects performed by the object detector. At the same time, the immediate input to the model is the set of visual object features, and learning similarities and differences between these features has made models perform so well on the number of visual and multi-modal tasks, e.g. image classification (Krizhevsky et al., 2012). Thus, we examine if semantically similar objects also share similar visual features produced by the object detector. We calculate the cosine distance cos_*vis*_ between feature vectors of objects within the same thematic cluster and objects from two different clusters, following the :

$${cos}_{vis}=\frac{\;\;\;\;\;v_{i}\;\;\;\cdot \;\;\;\;{v_{j}}^{⊤}}{\left\|v_{i}\|\cdot \|v_{j}\right\|},$$ (7)

where **v**_*i*_ and **v**_*j*_ are two feature vectors from the set of visual features **V**, belonging to either the same thematic cluster or two different clusters for a specific image. We first averaged the scores across all combinations of object features, then across all combinations of clusters, and, finally, across images. We ignore clusters that consist of a single object. We found that the visual features of objects placed in the same thematic cluster are more similar to each other (0.50) than the objects in two different clusters (0.31). This finding indicates that semantic similarity entails visual similarity, as has been observed in the learning behaviour of both humans (Rosch, 1975, 1978) and machines (Deselaers and Ferrari, 2011). This result can be attributed to bounding boxes in the input, which are in either part-whole relations and tend to largely overlap (e.g., “leg” and “gray cat”) or visually similar (e.g., bounding boxes of two cows in Figure 4). Thus, in earlier layers, the model links thematically related (semantic bias) and visually similar objects (visual bias). Deselaers and Ferrari (2011) also illustrate that distant objects are semantically less similar, while visually close elements are more similar to each other. In the following experiment, we examine if a similar bias is observed in our model based on the attention links of the image encoder.

### 3.2 The Effect of Geometric and Thematic Biases

In this experiment we inspect if there is an association between the number of attention links relating object pairs and the distance between the centres of these two objects. We make an analysis for objects in the same vs different thematic clusters. The center coordinates of each object (ObjCent_*x*_ and ObjCent_*y*_) are calculated according to Eq. (8), where *x*_min_ and *y*_min_ stand for the bottom-left coordinate point of the 2D bounding box covering the object, *w* is width, *h* is height. We calculate the Euclidean distance between two points in terms of image pixels. Attention weights are taken as they are without any modification.

$$\begin{array}{c} {ObjCent}_{x}=x_{\min}+w/2\\ {ObjCent}_{y}=y_{\min}+h/2 \end{array}$$ (8)

**FIGURE 6:**
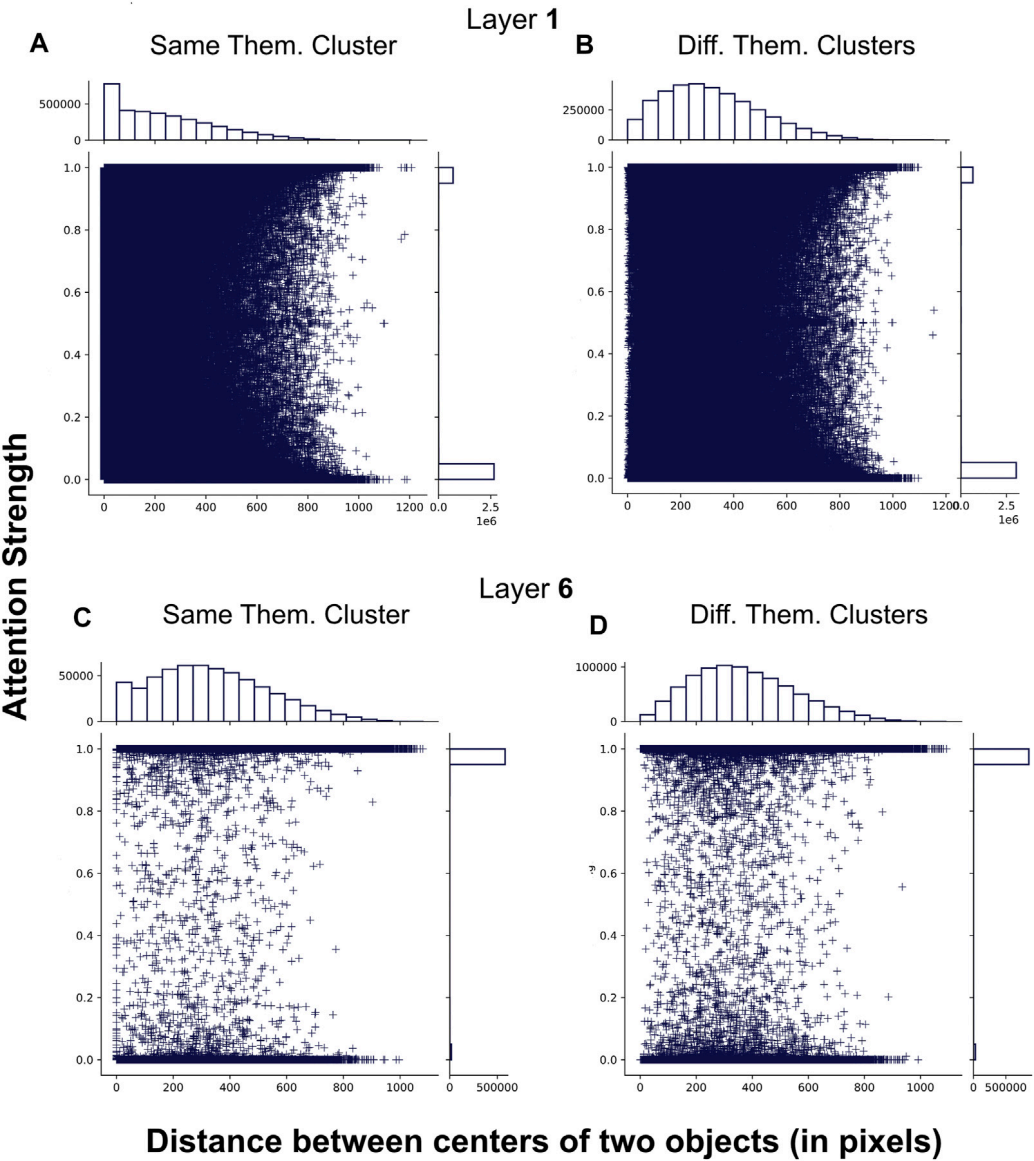
Four visualisations portraying the distribution of attention links (depicted as +marks) in terms of their strength (the vertical axis) and distance between centres of two connected objects (the horizontal axis). We split attention links into two groups: those between the objects that are either in the same or a different thematic cluster. **(A)** and **(B)** demonstrate the patterns observed in the first layer, while **(C)** and **(D)** demonstrate patterns in the last layer of the image encoder. A marginal histogram accompanies each visualisation for both the vertical **(top)** and the horizontal **(right)** axes. The histograms show a distribution of +marks for each dimension (either horizontal or vertical) in the scatter plot defined by a maximum of 20 bins. The scales for the bar sizes (e.g., frequencies of marks in each bar) are shown on the left side of the horizontal histogram and below the vertical one.

Figure 6 shows the distribution of attention links for different configurations of layers and thematic clusters in terms of attention strength (e.g., high or low attention weight) and distance between two objects. The left-skewed pattern in the marginal histogram for the horizontal axis of Figure 6A shows that more than 500, 000 of the attended and thematically related pairs of objects are close to each other ( ∼50 pixels). While there are still many thematically related objects that are not next to each other (e.g., numerous + in the range between 200 and 600 pixels), the majority of the related objects are immediate neighbours of one another. For example, in Figure 2 the centre of the bounding box of “grey cat” is 105 pixels away from the centre of “brown ear” and 219 pixels from “leg”. A gradual and stable left-to-right decrease of the bars’ sizes in the horizontal histogram is followed by an increasing distance between the objects on the horizontal axis, indicating that this layer learns a direct association between visual proximity and semantic similarity of objects. In comparison, we see fewer links between adjacent objects in Figure 6B since according to the horizontal histogram on top of the sub-figure, there are less than 250, 000 links between objects in close visual proximity ( ∼50 pixels), which is much smaller than the number of the closest thematically related objects observed in Figure 6A. Also, most of the thematically non-related objects are at least ∼200−300 apart. Note that the decreasing frequency of marks is accompanied by the increase in distances between paired objects. However, this decrease starts with the objects approximately ∼300 pixels away from each other. The pattern for thematically unrelated objects is very similar in the last layer (Figure 6D). While the overall number of links between such objects is much smaller (888, 103 vs 3, 773, 134 in the first layer), the vast majority of the objects ( ∼100,000 ) are nearly 300 pixels apart.

On the other hand, in the last layer (Figure 6C), the pattern observed for thematically related objects is different from what we have seen in the first layer. There are dramatically fewer links between neighbouring objects ( <50,000 ). Most objects are also more distant from each other, between 200 and 400 pixels. The last layer builds only 600,233 links between thematically related objects, while the first layer learns 3,439,620 such links. These differences indicate that the first layer might contain links between numerous objects in part-whole relations, where parts are both thematically related and visually close to each other, e.g., “leg” and “grey cat” in Figure 2, while the last layer learns thematic relations of a different kind with fewer objects, which are in a larger distance from one another, e.g., bounding boxes of two cows in Figure 4.

We used the non-parametric independent Mann–Whitney *U* test (Mann and Whitney, 1947) to examine if the differences in distances between attended objects are statistically significant between four different sets of attention links in Figure 6 (A, B, C, D). In this experiment, we refer to distances between objects according to the label of the corresponding sub-figure in Figure 6. For example, A refers to distances between thematically related objects as captured by the first layer. We found that the differences between combinations of all four sets of distances are statistically significant with extremely large *U* ^5^ and *p* consistently being 0.0. Such behaviour can be attributed to the size of our sets since it has been shown that statistical tests suffer from diminishing *p*-values when the size of the samples gets bigger, and even slight differences between large groups are considered significant (Lin et al., 2013). For example, A and B have more than 3,000,000 elements, while C and D have fewer items (600,000), but these numbers are still very large. Thus, we also compute Cohen’s d (Cohen, 1988) to measure the effect size between two populations to estimate the degree of differences, which might give us the indication of significance. The test has shown that the effect size of significance is medium between A-D^6^ (*d* = 0.701), small for A-C (*d* = 0.494), A-B (*d* = 0.423) and B-D (*d* = 0.292), very small for C-D (*d* = 0.182) and B-C (*d* = 0.096). This result demonstrates that, in general, distributions seen in earlier layers are different from those in the deeper layers. Intra-layer, the differences are insignificant for the last layer (C-D) and somewhat significant for the first layer (A-B). In addition, the difference between set A and other sets is more significant than all other set combinations. Therefore, we argue that local relations between semantically similar objects are captured in the first layer, less so in the last layer. Overall, the results show that the layers of the image encoder capture different kinds of knowledge, supporting the hypothesis of separation between learning of local and global dependencies in early and deeper layers of the model, respectively.

**FIGURE 7:**
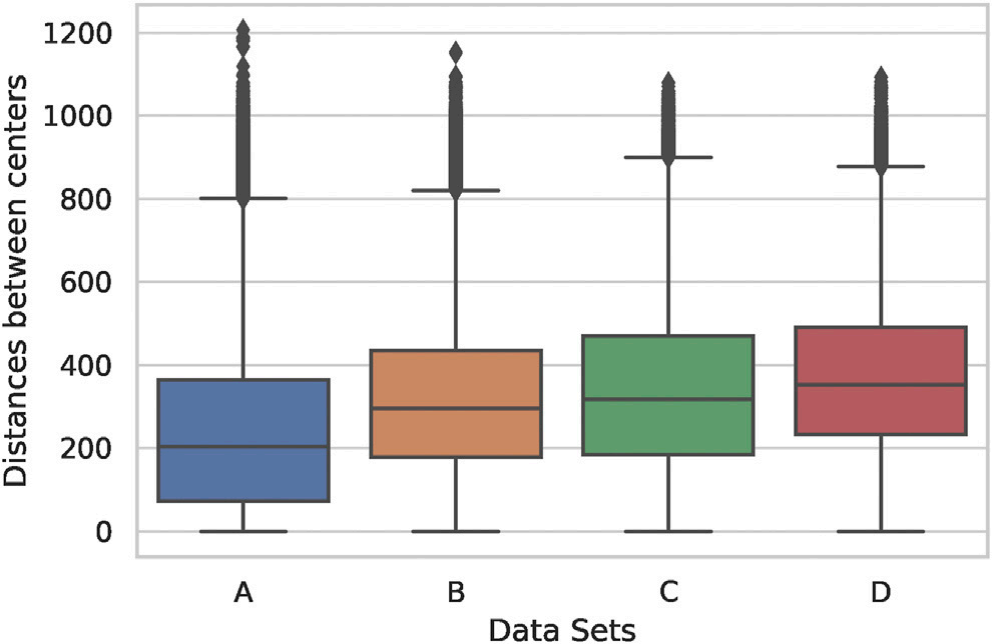
Box plots of distances between attended objects depending on thematic relatedness and depth of the layer. The vertical axis has measurements in pixels. Names of the data sets correspond to the conditions described in Figure 6.

In Figure 7, we provide additional analysis of the data and show the box plot of distances between attended objects for different distributions. The distance between 50% of all objects across all data sets (as witnessed by the quartiles) is lower than 500 pixels, while the outliers are nearly 1,000 pixels away from each other. The data in A is skewed to lower distances more than in other sets, supporting the idea of learning of local dependencies of thematically related objects in the first layer. This result suggests that the model links two objects that are both approximately in the image’s central area, not on the periphery. Intuitively, such learning mirrors the perspective of how the pictures are typically taken, e.g. most of the salient objects are usually distributed in the centre of the image, not around its corners. However, there are numerous outliers in all four sets, indicating, possibly, links between false object detections or simply non-informative links. Also, outliers might indicate such objects in images that are far away from each other but still belong to the same thematic cluster, as is the case for A. Additionally, the outliers in terms of distances between attended objects might generally occur due to the ability of self-attention to attend across all objects in the scene simultaneously. The outliers in the first layer are more spread and distant from each other, reaching differences as much as 1,200 pixels, compared to the last layer. At least half of the objects in C and D (two quartiles representing each box) are also more distant than in A and B. This finding indicates that deeper layers focus on more distant objects but not on objects that are extremely far from each other. Overall, the box plot shows that the first layer learns dependencies between neighbouring objects and a general understanding of the scene, attending between several very distant objects. In contrast, the last layer expands its focus across more distant objects and shrinks its scene-level attention, linking fewer objects on different ends of the image.

We also examine whether the strength of attention is significantly different across all four plots shown in Figure 6. We used the Mann-Whitney *U* test and found that the differences between all sets of attention strength are significant^7^. As we previously argued, significance testing for large data sets has to be accompanied by measuring the effect size to decide if the difference can be neglected. We used Cohen’s d and found that the effect size of significance is large between sets from the first and the last layer (A-C: 2.447, A-D: 2.381, B-C: 2.661, B-D: 2.586) and very small between sets within the same layer (A-B: 0.073, C-D: 0.042). This result shows striking differences between attention strength observed in earlier and deeper layers. For example, in Figure 6 the strength of the attention links ranges from 0 to one in the first layer. Most of the links are weak, as indicated by the marginal histogram on the right side of both sub-figures. In contrast, the deeper layers are generally more confident in connecting pairs of objects as most connections are close to the maximum strength (1.0). One possible explanation for such difference is that the model operates with original features of objects in lower layers and, thus, builds a large number of attention links of various strengths. In comparison, at higher layers, the representations are built on top of the information from lower layers and might closely correspond to the conceptual knowledge of objects. Therefore, we observe more confidence in the attention patterns in deeper layers rather than in earlier layers.

**FIGURE 8:**
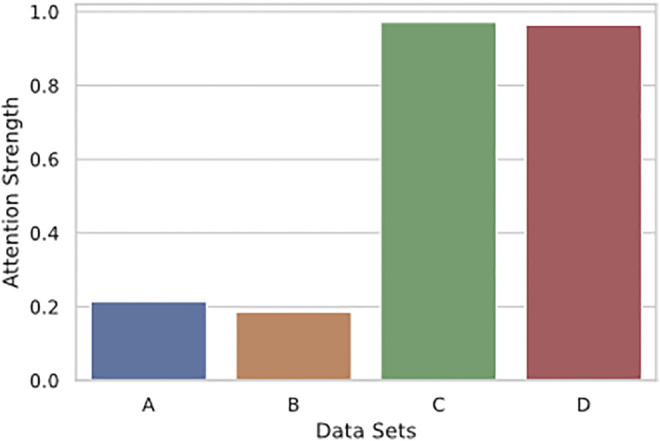
Bar plot that shows means of the attention strength in each of four conditions. The *y*-axis describes the strength of the attention, ranging from 0.0 to 1.0. Names of the data sets correspond to the conditions described in Figure 6. Standard deviation for different sets: SD **(A)** = 0.406, SD **(B)** = 0.384, SD **(C)** = 0.164, SD **(D)** = 0.184.

In Figure 8, we provide a bar plot that visualises differences in the attention strength of links built in the first and the last layer of the model. The figure caption also describes standard deviation (SD) of values in each set. In earlier layers, the model has very diverse attention based on the SD values, its mean is relatively small ( ∼0.2 ). Attention in deeper layers has much smaller SD values, and its mean is nearly 1.0. These results additionally supports the idea that deeper heads are more focused on specific relations, while in earlier parts of the model the attention is distributed across many objects.

Overall, we have found a noticeable and statistically significant difference in the knowledge captured by earlier and deeper layers of the image encoder. The first layer, in particular, captures a general understanding of the scene by linking neighbouring objects with low certainty (small attention strength). In contrast, the last layer is highly confident in linking objects, additionally spreading its attention across more distant objects. There is also a difference in the thematic knowledge captured between the layers: the first layer might acquire information about the thematic relatedness of objects in local dependencies (e.g., part-whole relations). In contrast, the last layer broadens the notion of thematic relatedness and capture similarities between whole entities in larger distances.

### 3.3 Knowledge Split Between Self-Attention Layers

We also examine the differences between layers in terms of the similarity of the corresponding weights with each other. Specifically, given the previous experiments on differences between the first and the last layer only, we want to inspect where (between which layers) the shift in the learned knowledge happens in the image encoder. Following Zhou et al. (2021), we compute the cosine similarity between attention patterns observed in two neighbouring layers for all images in the test set. In particular, for every object *n*_*i*_ in the image, we compute cosine score between two vectors representing attention originating from this object to every other object in the image at layer *k* and layer *m*:

$${cos}_{h,n_{i}}^{k,m}=\frac{\;\;\;\;\;\;A_{h,n_{i}}^{k}\;\;\cdot \;\;\;\;{A_{h,n_{i}}^{m}}^{⊤}}{\left\|A_{h,n_{i}}^{k}\|\cdot \|A_{h,n_{i}}^{m}\right\|},$$ (9)

where $A_{h,n_{i}}^{*}$ is the self-attention vector for a specific object, *k* and *m* are two neighbouring self-attention layers (e.g., layer three and layer four), and *h* is the attention head. The final cosine similarity scores for each attention head are averaged over all objects (divided by *N*). We also average the scores over heads and images to obtain a single score per pair of layers. Here, we examine the similarity between attention patterns of *neighbouring* layers only since we want to inspect how visual knowledge is sequentially processed from earlier to deeper layers.

**FIGURE 9:**
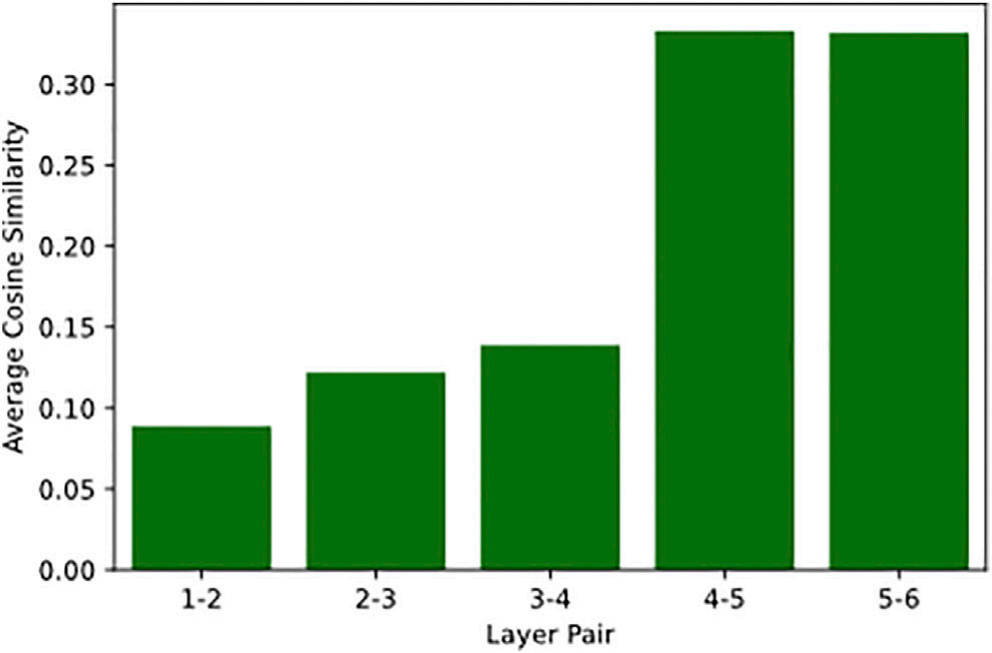
The average cosine similarity between attention weights in neighbouring layers. The horizontal axis shows the specific pair of layers that the scores were calculated for, while the vertical axis shows the cosine scores.

Figure 9 shows the results. The attention patterns are highly dissimilar in the first three layers. In contrast, deeper layers (4, 5, 6) encode more similar knowledge, showing an increase in similarity by almost 0.25 points. This indicates that the results of the analysis in Section 3.1 and 3.2 can be valid for the layer four and five because of their strong similarity with the last layer. At the same time, the dissimilarity between the first three layers can be explained based on what we know about the first layer, which builds many different links of various strengths between the objects. Thus, it is possible that layers two and three also build a large number of varied links of different strengths. In general, the results suggest that the shift in similarity occurs somewhere in the middle of the image encoder. Such change can be attributed to the type of information each layer operates with and how.

We also examine the level of dispersion of attention links in different layers of the model and compute the attention entropy *E* of the attention distribution *α* for each attention head according to the Eq. (10), where *s*_*i*_ and *t*_*j*_ are specific source and target objects, *α* is the attention value between them.

$$E_{\alpha_{\ell ,h}}\left(t_{j}\right)=-\overset{\left|S\right|}{\underset{i=1}{\sum }}\alpha \left(s_{i},t_{j}\right)\;\log\left(\alpha \left(s_{i},t_{j}\right)\right)$$ (10)

**FIGURE 10:**
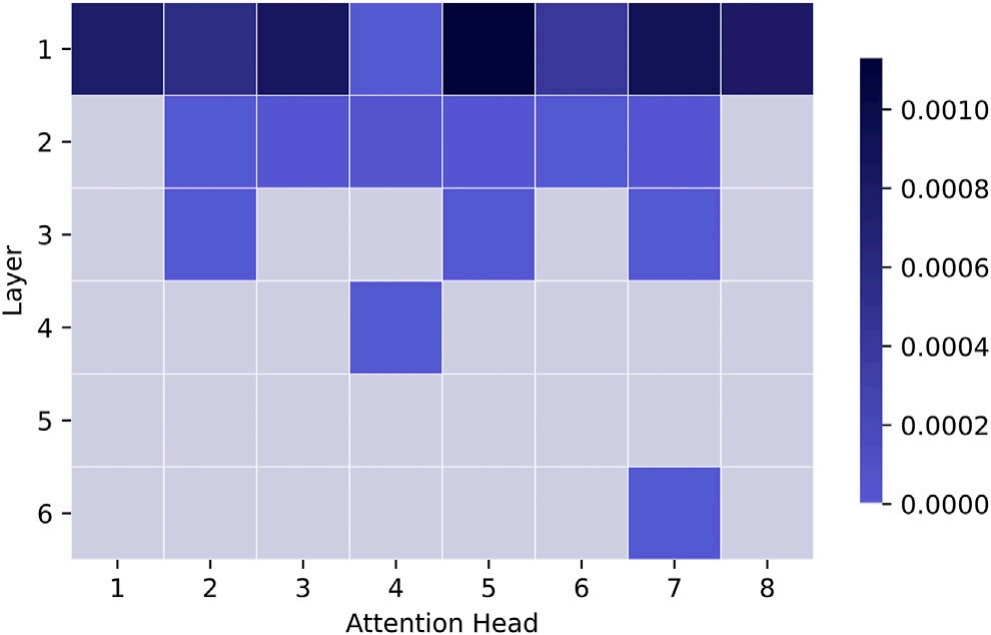
We show the normalised entropy of visual self-attention heads. The result has been normalised by the maximum attainable entropy log(*N*). The darker colour indicates higher entropy.

The results in Figure 10 show that all attention heads do not contribute equally to the formation of attention links. In general, the entropy is highest in the first layer and slowly diminishes with the depth of the model. This pattern demonstrates that attention converges to relate fewer objects in deeper layers compared to the earlier ones. Based on the results in Section 3.2, this knowledge is also established between more distant objects. This shows that the attention in deeper parts of the model becomes saturated and focused on specific relations, possibly reflecting the knowledge of core entities in the scene, perceived on the scene level, not local level. Overall, the image encoder captures at least two types of knowledge, which can be associated with either the first layers of the model or the deeper ones. Disperse and dissimilar attention in the initial layers indicates that the model starts with learning general and varied aspects of the scene. In contrast, highly focused and similar links in deeper layers show that the model converges to a more concrete understanding of the image, potentially establishing task-dependent relations between objects. At the same time, the representations in deeper layers are not extremely similar (e.g., they do not reach a 0.9 score or higher). It indicates that the attention patterns in these layers still show active learning of diverse connections between image objects and are less likely to encode any layer-redundant information. Note that it is much harder to establish strong attention links in earlier layers, given only a few steps of visual information processing in previous layers. The confidence of deeper layers, thus, might be not only built on top of the previous processing but also shaped by the task.

Eventually, the image encoder is required to produce valuable representations for caption generation, and the last layer outputs such representations. In turn, captions describe only specific relations between objects and not the whole set of all possible relations. Thus, the reasons for the presence of focused relations in deeper layers can be two-fold. First, the emergence of more concentrated knowledge can be due to the learning of complex information from low-level image understanding in the initial layers, as observed in the experiments. Second, indirect interference of the language task, leading to high-level conceptual knowledge of the scene in deeper layers, can also be an important factor. Representations from these layers are used as input to the cross-attention module, which fuses them with language representations to generate the image description. In the following experiment, we examine whether there is an association between the caption and representations from the last layer of the image encoder.

### 3.4 Tracing the Knowledge of Language in Visual Representations

Here, we examine if deeper layers of visual self-attention achieve a better pairing of **noun** phrases in captions, typically describing objects in relations on the global scale, with objects in the scene through the attention links. By inspecting whether visual representations include signs of conceptual (language) knowledge, we enrich our understanding of how different modalities affect each other in the multi-modal transformer (see our work in Ilinykh and Dobnik (2021) for the discussion on how language representations are affected by visual information).

Our goal is to compute the proportion of attention links, which mirror the pairing of objects and noun phrases in captions. In our analysis we name objects that receive attention as target objects, normally shown on the right side of attention visualisations (e.g., Figure 4 and Figure 5), while objects that are source of attention are named as source objects. Note that each target object might be connected with any other object in the scene, receiving attention from multiple source objects. This can lead to such attention links, which are hard to interpret since the target is attended by many different source objects. An example of this can be observed in Figure 5, Layer 3: the attention head represented in purple predicts the “motorcylce” as the target of attention for all the source objects on the left. Similar to *null attention* observed in the analysis of textual models (Vig and Belinkov, 2019), we treat such attention links as non-informative. Specifically, if a target object is linked with more than 30 source objects, we ignore all such links for the current attention head.

Next, we prepare the set of noun phrases and object labels for linking. We use spaCy^8^ to identify and extract noun phrases from image captions. Since the labels of the detected objects describe specific *objects* (e.g., “a cat”) rather than other parts of the scene (e.g., “the right corner”), we perform additional filtering of noun phrases. We remove any phrase which contains at least one word from the special word list^9^. We keep adjectives in noun phrases since object labels are typically provided with attributes. Note that numerals and determiners are removed from the noun phrases in order to reach structural similarity with object descriptions for the linking process. After obtaining the set of noun phrases from the caption, we process the detected objects to collect their labels. For every object, we retrieve the predicted label (noun) and its attribute (adjective). The attribute is removed if the extractor is not confident about the attribute’s correctness, when the confidence is lower than 0.1.

We want to inspect whether the linking between the target object and source objects corresponds to *two different* noun phrases, which are also related, but in the context of image description. According to the attention links between objects (e.g., Figure 4), a target object (the right side of visualisations) is often linked with multiple source objects (the left side). While linking one object label depicting the target with the noun phrase is relatively simple, connecting multiple source objects with a single noun phrase is not straightforward. Objects might be associated with more than one noun in the caption, and the other way around, a caption might be related to more than one visual entity in the image. We take a simpler approach and inspect if at least a subset of source objects for a specific target can be grouped into a single thematic cluster, thus, describing a single entity. By identifying from all the detected objects those representing the core scene entities, we ensure that the large part of different links captures identifiable relation for a single target. For example, if many source objects can be associated with a single entity that is linked with a different entity depicted by the target (e.g., the target “cow” is attended by a large subset of source objects which form a thematic cluster of “building”), the attention head can be interpreted to be confident in linking these two entities. In contrast, highly diverse links between target and source objects (e.g., the target “cow” is linked by several kinds of objects representing “building”, “trees”, and “street”) leads to a weaker knowledge of specific relations in the scene since no semantic category is dominating the weights. Such a spread of focus might lead to less interpretable and diluted knowledge. To distinguish between confident and non-confident patterns of attention, we cluster source objects based on the same strategy we used in the first experiment (Section3.1). In particular, we impose a relatively soft requirement and examine if at least 25% of the source objects can be grouped into a single thematic cluster. We ensure that this cluster is different from the cluster of the target object. If we can not group at least a quarter of source objects’ labels into a single cluster, we ignore the whole set of links for this particular set of source objects and a target. This simple mechanism ensures that we select only those attention links between objects that are strongly focused and connect entities from two different thematic clusters.

Once the objects are clustered, we match their labels with noun phrases by computing their semantic similarity score. We use BERTScore^10^ (Zhang et al., 2020) to get the most semantically similar object label for every noun phrase. This model allows us to use the power of pre-trained contextual BERT embeddings (Devlin et al., 2019) to match each noun phrase with every object description and the other way around by computing cosine similarity. The model also correlates well with the human judgements and outputs results of multiple performance metrics: precision, recall, F1 score. A recall metric is computed as the averaged sum of maximum cosine similarities between each token in the noun phrase and a token in the object description. Precision is calculated similarly, but between each token in the object description and a token in the noun phrase. F1 score is a classic combination of precision and recall. In the end, we receive a single F1 score for every combination of a noun phrase and the whole set of object descriptions, which can be either targets or sources of attention.

We match the source objects and noun phrases as follows. First, as described previously, we take the subset of object descriptions if one-fourth (the size of the subset) of the whole set can be grouped into a single cluster. F1 scores of the nine objects in the subset (25% from *N* = 36) are ranked from highest to lowest for each noun phrase. We examine if there is an F1 score higher than 0.6 in at least *one* of these subsets of source objects to ensure that the word similarity scores are sufficiently high. If that is the case, a set of nine objects is linked with the noun phrase that has the highest similarity score with this set compared to other noun phrases. For example, “a cat” will be chosen as the linked noun phrase for nine source objects if their highest F1 score is 0.8 for the object description “white cat” from this set, compared to a different noun “the table”, for which the highest F1 score (0.6) for an object description from the set is smaller. The target object’s label is inspected for the biggest F1 score across all noun phrases and matched with the noun phrase with an F1 score higher than 0.6.

Given two noun phrases describing the source and target objects, we check if these two phrases are different. If these phrases are different, we proceed to calculate the proportion of attention links between mapped objects vs all objects for all images and target-source connections. Note that if we were able to successfully map nouns with object descriptions, the computed proportion for the specific target is Prop_*target*_ = 9/36 = 0.25. We get the final results by averaging these proportions across all targets and images.

**FIGURE 11:**
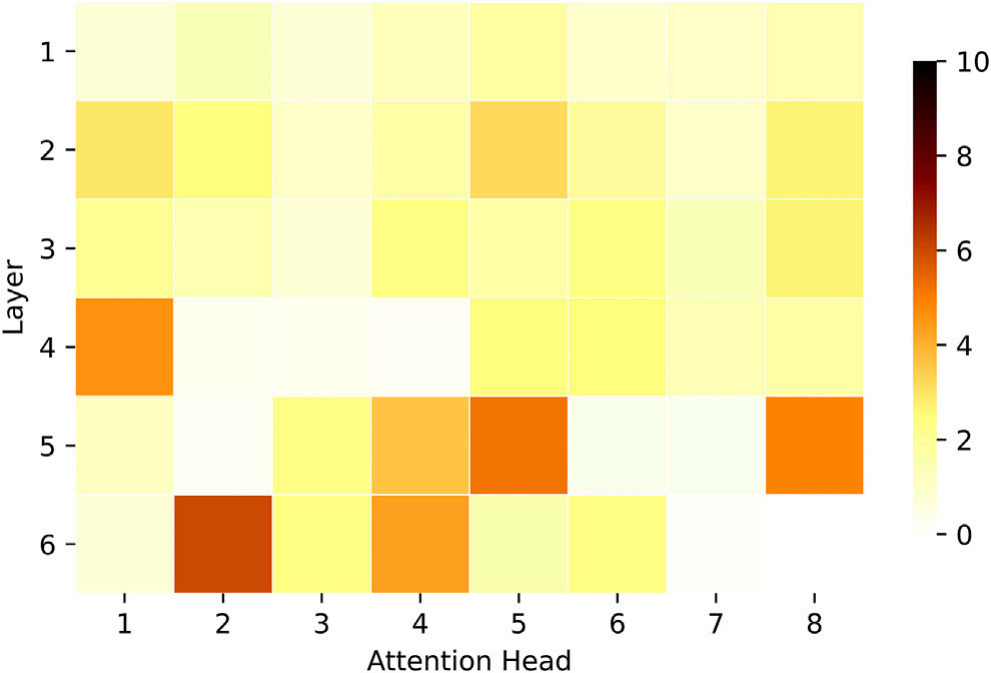
The proportion of attention links between target and source objects which can be associated with at least one noun in the caption. The nouns cannot be identical (e.g., “cows” and “building” in “two cows outside, one laying down and the other standing near a building”), and both the target and the source objects linked with them must correspond to two different thematic clusters. For better visualisation, the highest value is set to 10%.

The results are shown in Figure 11 in percentages. The knowledge of relations between two different entities mentioned in the caption and expressed as linked objects is more pronounced in deeper layers of the model. In fact, Figure 3 shows that deeper attention heads are more active when connecting objects in different thematic clusters, which possibly correspond to two different noun phrases. These objects describe entities that constitute the visual scene as a whole and are also more distant from each other (Figure 6B). For example, in Figure 2 the “grey cat” is 305 pixels away from the “yellow banana”, which is much further than the distance between the vast majority of semantically similar objects ( ∼50 pixels, Figure 6A). In addition, the caption for this image (“a cat that is eating some kind of banana”) describes it with global relations between objects, not with local, thematic relations (e.g., “a cat with paws and a tail is eating some kind of banana with a yellow peel”). The structure of image descriptions depends on the pragmatic context of the task and instructions given to the describers. In the case of the MSCOCO dataset, the instructions forced the participants to describe the image as a whole with a single sentence. These regulations have primed humans to mention only a subset of the most important objects in the scene, leading to 11.30 words per sentence on average, according to the statistics of MSCOCO dataset. We have identified that such knowledge of described objects is concentrated in deeper attention heads, indicating the traces of language information in visual representations. This finding also corresponds to the idea that deeper layers provide cross-attention with the necessary task-dependent representations, encoding more global knowledge of the scene in terms of whole entities (not numerous detections of objects of smaller granularity)^11^.

Note that the attention heads in the first layer have bright uniform patterns, indicating lesser mapping of nouns in captions and object descriptions. We attribute such knowledge to the design of self-attention, which might somewhat understand relations between objects on the scene level in the initial layers. However, this knowledge is less expressed in the first layers and more articulated in deeper layers. Besides, the patches for multiple heads in deeper layers are nearly entirely white. We believe that these heads might also capture mapping between nouns and objects but in terms of a different and hypothetical image description. For example, one can describe images in various ways (e.g., each image in MSCOCO comes with five descriptions). Figure 2A can also be described as “a cat is on the white floor” or “someone is feeding a cat with a banana in their hand”. In these texts, the focus is on such image entities that differ from those mentioned in the original caption (“a cat that is eating some kind of banana”). It is possible that due to the nature of self-attention, deeper layers were able to capture these potential relations between different objects as well, given that the model was provided with all five captions for each image during training. In addition, similar entities co-occur across images in different conditions and configurations. This type of knowledge might give self-attention even more power to juggle the observed objects in myriad ways. However, an additional set of experiments is required to unveil what else is learned at the output of the visual self-attention, and we leave this for future investigation.

### 3.5 Representing Image With Patches, Not Objects

In our experiments, the model attends across the visual features of bounding boxes, which correspond to objects in images, providing the model with semantic information about images. Such representations have shown to be more suitable for image captioning and visual question answering (Li et al., 2017; Anderson et al., 2018). However, more recently, dividing images into a uniform grid of patches of the same size and feeding them to a BERT-inspired visual transformer has demonstrated improvements on the task of image classification Dosovitskiy et al. (2021). Note that prior to their application in vision transformers, grid-like representations have been widely used in encoder-decoder networks for image captioning (Xu et al., 2016; Lu et al., 2017), but were shown to be less informative than object-level representations.

Here we perform an ablation study, examining the distances between attended objects in two settings: 1) when the model is given grid-level features (image patches), 2) or when the model uses object-level features. Note that we used object-level representations in all other experiments in this manuscript. We divide each image into the set of 6 × 6 patches, resulting in 36 patches per image, comparable with all our experiments in which we detected 36 object regions. Next, we pass image patches through ResNet-101 (He et al., 2016) and extract the set of patch-based visual features **P** = {**p**_1_, … , **p**_*N*_}. These representations are used as an input to the self-attention layers similar to linearly projected patch embeddings in Dosovitskiy et al. (2021). Our feature extractor is fixed and not updated during training. We train the model from scratch on the set of image patches and the corresponding caption. We follow the instructions in the original paper by Herdade et al. (2019) and use the official code implementation^12^ to train the model in two stages: first, with a standard cross-entropy loss, then we use self-critical reinforcement learning (Rennie et al., 2017). The model is also provided with the relative positions of image patches. We test the model on the Karpathy test split (Karpathy and Fei-Fei, 2015) in a teacher-forcing setting and extract its attention links with the corresponding weights. The grid-based representations are not informed semantically; hence, they might not correspond to a semantically plausible object in the region. Therefore, we perform the analysis of distances between attended parts of the image identical to the geometric analysis in Section 3.2

**FIGURE 12:**
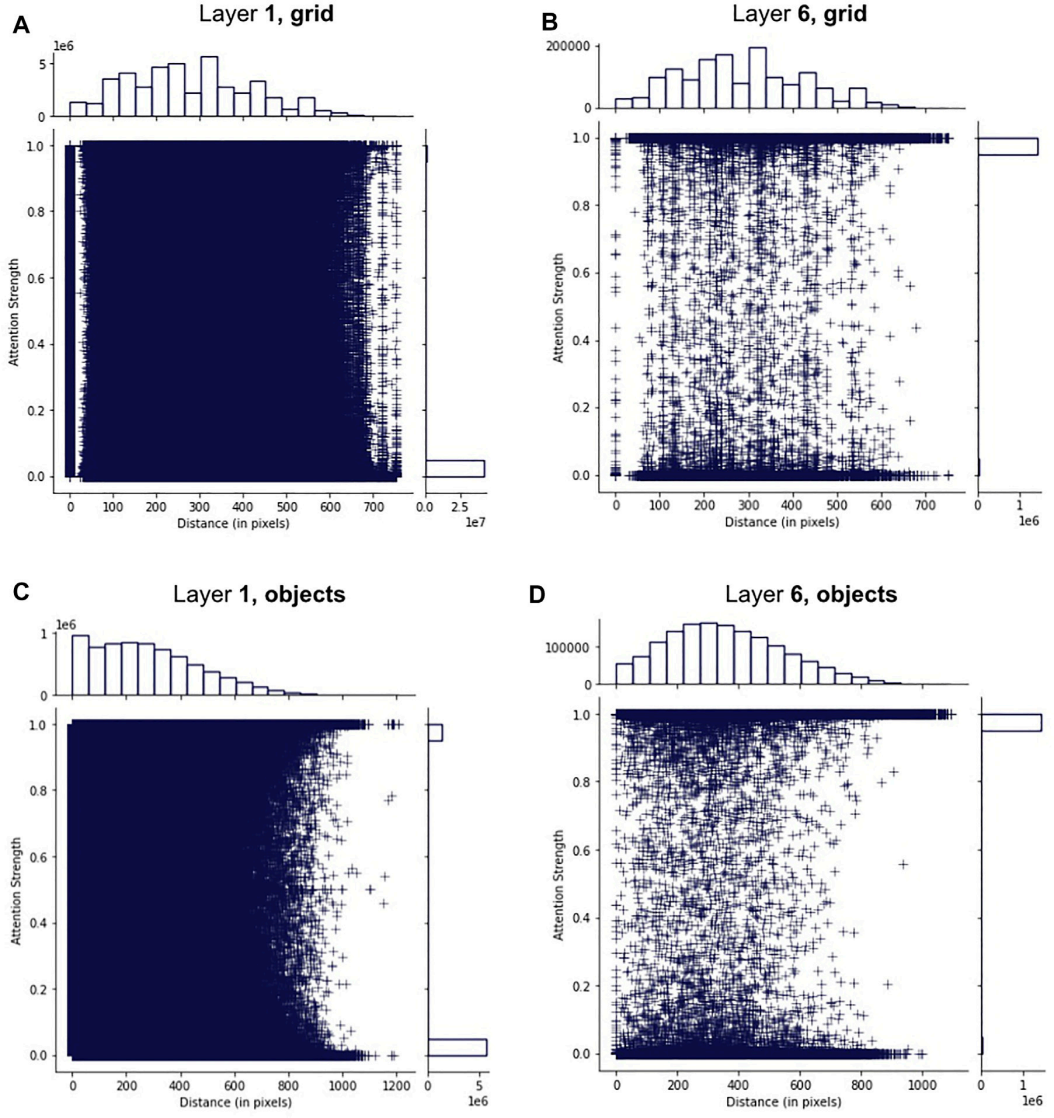
The distribution of the attention links (depicted as +marks) in terms of their strength (the vertical axis) and distance between centres of two connected objects (the horizontal axis). **(A)** and **(B)** correspond to the patterns from the first and the last layer of the model for grid-based features (e.g., image patches), while **(C)** and **(D)** represent links when we use object representations as input features. We disregard objects’ thematic clusters in these visualisations for a fair comparison with the grid-based approach. Information about other parts of the figures (e.g. histograms) is identical to the description in Figure 6.

Figure 12 shows the distribution of attention links when the model is provided with either image patches (A, B) or object representations (C, D). We focus on the analysis of distances between attended objects, similar to our experiment in Section3.2. First, note that the two distributions of attention links between layer one and layer six for the grid-based approach are visually very similar (A and B, histograms on top). We compute Cohen’s d score to measure the effect size between the observed populations to get the estimation of the degree of differences since a large size of the data makes results of significance testing non-informative, which has been observed in Section 3 2. Our results have shown that the effect size is small for A-D (*d* = 0.489), B-D (*d* = 0.422), and C-D (*d* = 0.402), very small for A-B (*d* = 0.082), A-C (*d* = 0.030), B-C (*d* = 0.039). Layer-wise, we observe that object representations affect the structure of the learned information in earlier vs deeper layers, which is reflected in a more noticeable difference between C-D, while this difference is practically absent between patterns in A and B. This leads to the conclusion that semantic information indeed provides *more structure* to what and how the model learns. Also, the distances patterns that are learned with grid-based features have not shown a large effect size of significance when compared with the patterns captured in the *first* layer of the object-based approach (A and B vs C). In contrast, the effect size becomes bigger when comparing patterns in both A and B with distances in the *last* layer of the model that uses semantically informed representations (D). Combined with the results in section 3.4, we conclude that there is no clear distinction between local and global knowledge in layers of the model when it is provided with image patches. Overall, our ablation experiment has shown how semantic information allows the model to organise the information that it learns hierarchically. We note that our results echo what has been observed by Raghu et al. (2021), who have shown that visual transformers that operate with image patches (aka grid features) do not structure their knowledge in the context of the image classification tasks.

## 4 Discussion and Implications

**FIGURE 13:**
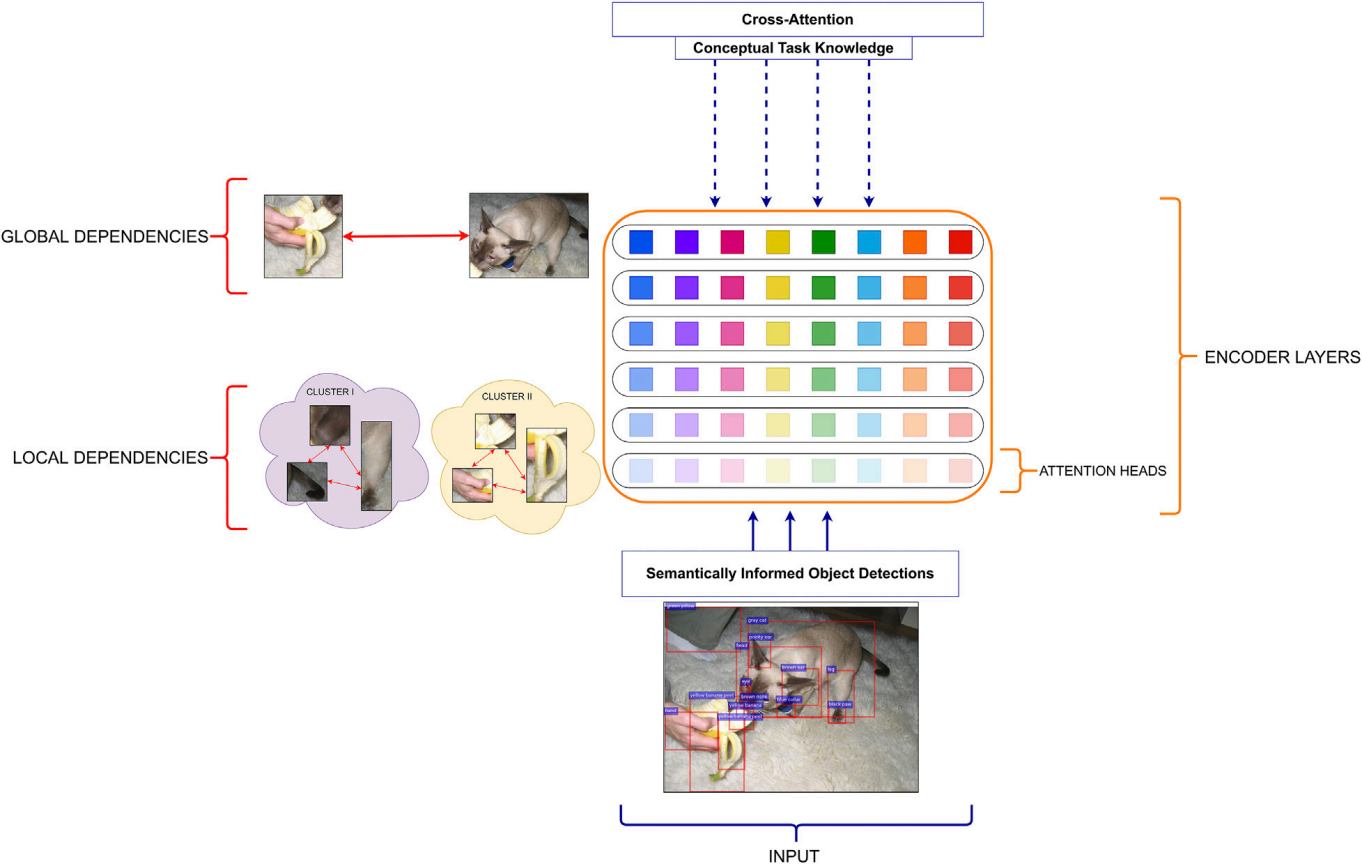
A visual conceptualisation of the most important findings of the current study. The colour intensity of each attention head increases with layer depth, resembling the increase in attention strength. Left: as shown, earlier layers capture more local dependencies between thematically related objects, while deeper layers connect distant objects. Below: the input to the model is the set of *semantically informed* features of detected objects, which is in contrast to the grid-based approach, where image patches carry no semantics. Above: we also denote an indirect influence (dotted lines) from the cross-attention module, which possibly occurs due to backpropagation and is reflected in the heavier grounding of noun phrases in deeper heads that we examine in Section 3.4. Overall, this figure demonstrates the whole variety of findings that we present in this manuscript.

Our primary goal in this paper was to identify what kind of representations are learned in the vision stream of the multi-modal transformer. Specifically, we have examined how different parts of the visual self-attention learn to attend between objects of varying granularity. In addition, we have also inspected if visual representations contain artefacts of world knowledge (language). Our model benefits from higher-level object detections and builds a variety of relations between *objects*, in contrast with the vast majority of vision transformers that receive pixel-level image representations as their input and lack grounding in richer language representations. In addition, the CNN-based object detector produces numerous bounding boxes, frequently corresponding to what humans consider parts of a single object. This, in turn, affects the type of knowledge our model captures or “sees” when attending across objects. The effect of using semantically informed visual representations has been validated by the ablation experiment in Section 3.5. Figure 13 illustrates how all of the results reported in this paper can be placed in a single visualisation. We conclude with the following results:

• Thematic analysis of objects based on their labels has shown that lower layers more frequently form attention links between objects, which belong to the same thematic cluster.
• Analysis of distances between attended objects demonstrates that objects in the same thematic class are also closer together. Thus, lower layers encode knowledge of local dependencies between objects, which might correspond to part-whole relations.
• The attention strength increases in deeper layers and stays at much lower levels in the earlier layers. This finding reflects gradually increasing confidence of the model about the objects that it has to relate for a better understanding of the whole image.
• One important finding is the effect of the input representations on how and what visual self-attention learns. We have demonstrated that higher-level object detections structure the internal representations of the model, while patch-based representations do not inform the model about object semantics; hence, there is no gradual learning from local to global knowledge.
• Lower layers compared with each another lead to very dissimilar attention patterns; higher layers exhibit more similarities. This result shows a shift in knowledge between the layers, with deeper ones being more specialised.
• Larger entropy in the earlier layers indicates their dispersion and attention between many different combinations of objects. In contrast, deeper layers are much more confident and focused on individual objects, indicating learning of saturated information about relations.
• Lastly, matching attended objects with noun phrases in image descriptions indicates that objects referred to in a description are more often attended in the deeper layers. This result shows the indirect influence of language on the model’s visual representations in the context of the image captioning task.

The first conclusion that we make here is that visual information is built-up hierarchically, starting with learning of local dependencies between objects and finishing with more global understanding of the scene, also in terms of the described objects. Similarly, structured representation learning has been observed for linguistic information, starting with more local dependencies in earlier layers and expanding to the global dependencies (e.g., subject-verb agreement) in deeper layers of BERT (Jawahar et al., 2019). In the beginning, the multi-modal transformer connects objects which are visually, semantically and locally close to each other and expands onto learning relations between more distant objects, collecting information about the image as a whole. In earlier layers, the model builds many links of lower strength between objects. In deeper layers, the model is re-distributing its attention towards fewer objects, but with much more confidence, reflected in larger attention strength. The deeper layers are affected not only by local visual dependencies from previous layers but also by the pragmatic nature of the image captioning task. In this task, there is a preference for depicting global scene-level relations in the caption, as participants were instructed to describe a scene with a single sentence. At the same time, all modalities are a part of a system as they are trained together, and therefore, one does expect their co-influence. Both language and vision information might optimise each other because the model needs to find a sensible mapping of one to another. Thus, due to the back-propagation of the information in the model (from caption to cross-attention to visual self-attention), deeper layers of visual self-attention might be indirectly guided by conceptual knowledge. At the same time, as our experiment in section 3.5 has shown, the structure of the learned knowledge is directly affected by the type of input representations (e.g., image patches or semantically informed object detections) given to the model.

The hierarchical processing of visual information has also been observed in humans. Our results recall the results of Hubel and Wiesel (1959): visual information is processed hierarchically with simpler biological cells responding to such phenomena as light orientation and more complex cells capturing movements. However, we can build a stronger parallel between our results and the theory of visual routines (Ullman, 1984). According to this theory, humans process visual information in sequential order, starting from more straightforward representations and later applying task-dependent rules to them. In particular, *base representations* are such features, which depend solely on visual input and typically are 2D image patches or sketches. Such features encode basic information about the scene: depth, colour and orientation. They are uniformed and bottom-up driven. As the next step, *visual routines* are applied to base representations to produce more complex features. Visual routines, in turn, are divided into a two-step process: in the first step, *universal routines* are used to achieve some general understanding of the scene. universal routines constitute the set of rules that allow us to perform some initial analysis of the scene and capture general aspects of the scene to isolate objects and describe their colour, shape, and other characteristics. Such routines are not task-dependent, and they are required to define such information, which more complex visual routines can later use. For example, universal routines might provide us with sufficient information to classify an image as a whole with objects in it. Finally, given the holistic understanding of the image from applying universal routines, we use more specific and top-down driven visual routines to complete the task at hand. When explaining visual self-attention and its processes through the prism of visual routines, it is possible to say that its input are base representations (object features), with different layers learning both universal and visual routines. In other words, the first layers of the model would mirror the behaviour of universal routines, capturing general information about the image. In contrast, deeper layers are specialised in building relations regarding the global arrangement of objects and their importance for the captioning task.

Moreover, humans learn about elements ‘below’ object level (e.g., the set of semantic and visual features corresponding to part-whole relations) and ‘after’ (e.g., identification of object relations) (Ben-Yosef and Ullman, 2018). This structure mirrors the process of learning of local and global information observed in visual self-attention. Local knowledge, in particular, can also be related to the innate ability of humans to represent objects through the hierarchy of their parts. The principle of compositionality can be observed not only in the language in the sense of Fregean tradition but also in human vision, e.g. computational vision (Geman et al., 2002). Parts often cannot be represented in isolation, without any contextual constraint on them, that would allow them to form a coherent whole. Consolidating parts into a whole is not task-dependent and can be seen as a bottom-up guided feature of human cognition. Yosinski et al. (2014) show that such basic abilities may be considered as general knowledge that is acquired in earlier layers.

We have also observed that the objects attended in deeper layers are more likely to be matched with noun phrases in image descriptions. This result suggests that conceptual (language) knowledge is indirectly present in visual representations of deeper layers. Here, we show that this result corresponds to the insights from a well-developed load theory of selective attention and cognitive control (Lavie et al., 2004). As this theory suggests, in terms of human cognition, attention acts through two selective mechanisms: *perceptual selection* and *cognitive control* (Dobnik and Kelleher, 2016). In the generation of image descriptions, both of these processes are required. In particular, describing an image is a complex cognitive task as the describer must select what information to include in the description. Using all perceptual and background knowledge, humans decide what objects they should mention. Thus, the perceptual selection is identical to filtering perceptual information by our sensors, while cognitive control is a selection of elements from this information given the task. In other words, humans cognitively control *when and how* to describe specific objects and relations between them, which are perceived and filtered visually. Note that the pragmatic nature of the image captioning task places strong restrictions on what is to be described. For example, in the task of image paragraph generation as Ilinykh and Dobnik (2020) note, there is a progression in image description from the general (“an image with two chairs.“) to more specific knowledge (“the chair on the left is black”). In this case, the restrictions on filtering visual scenes and controlling what to describe are not substantial since the model generates descriptions of a larger set of objects and relations throughout multiple sentences.

In our experiments, the model back-propagates representations from cross-attention to each uni-modal stream. The cross-modal representations include both vision and language information combined. Hence, we expect that there is an effect that task-dependent information has on visual representations. We attribute this effect to the mechanism of cognitive control, which influences visual representations in deeper layers to be more beneficial for cross-attention and, eventually, the task at hand (image captioning). At the same time, deeper representations are created from the lower-level knowledge of local object dependencies coming from earlier layers. In our experiments, we have observed that the number of attention links decreases with the increased depth of the model, and they also become stronger (more focused). We see parallels with perceptual selection in this effect: initially, the model constructs many different attention links and filters them layer after layer. Overall, we have provided a preliminary evidence that the representations in deeper layers are affected not only by visual information coming from visual input, but also by conceptual knowledge, that indirectly makes deeper representations to be more language-aware (Section 3.4). However, we leave a more detailed analysis for future work.

One important implication of our work is the effect that the structure of large-scale models has on the representations learned by different modules responsible for processing different modalities and their fusion. As all modules are trained end-to-end and optimised jointly, it becomes impossible to avoid information leaks from one modality to another. However, in multi-stream architecture, these effects can be seen and analysed in isolation for each modality. One benefit of using such representation is their indirect grounding in a different modality. For example, as we have revealed in this paper, visual representations alone contain perceptual knowledge about the scene, which is structured and partially organised by task-related language knowledge. Combined with the results in Ilinykh and Dobnik (2021), we argue that uni-modal representations resemble at least partial grounding in a different modality, which is just as good as a result of the cross-modal fusion of two modalities, that is often too complex to explain and utilise. We believe that an extensive set of experiments is required to examine if the training task and structure of the multi-stream transformers is the exact reason for such exciting blends of different modalities in a single modality’s representation.

## 5 Conclusion

A large number of papers has focused on the analysis of representations captured by uni-modal architectures, e.g. BERT (Devlin et al., 2019). This manuscript shifts the attention from uni-modal to multi-modal architectures and presents the analysis of visual representations learned by the two-stream image captioning transformer. We show that the visual knowledge is hierarchically structured as resembled by the self-attention weights of the visual stream. In particular, while earlier layers are better at learning the information about thematically related and visually close objects in the scene, deeper layers focus on objects that depict core entities on the image scale, capturing relations between them. We also demonstrate that the task affects the high-level knowledge in deeper layers, resulting in the artefacts of language found in visual information. We support our findings with several insights from the experiments in cognitive science. Overall, our extensive analysis touches upon fundamental questions on the effects of the model’s architecture and multi-modality on the model’s representations. We argue that representations of each modality can be enriched with important information from a different modality, which helps build more efficient and robust architectures. In future work, we are planning to test each of the three modules in the multi-stream transformer for several multi-modal tasks such as visual co-reference resolution and multi-modal human-object interaction.

## Data Availability

The raw data supporting the conclusions of this article, including the code, is available here: https://github.com/GU-CLASP/what-does-a-language-and-vision-transformer-see.

## Footnotes

1 Note that there is also an effect of how a target object is placed in the image. In standard image classification set-up, the target object is located in the centre of the image, intuitively simplifying the task for the model.

2
https://github.com/peteanderson80/bottom-up-attention

3
https://github.com/yahoo/object_relation_transformer

4 We use layer-head notation.

5 The U values were ranging from 237,983,526,041.0 to 1,391,402,498,035.0. Medians of the sets in pixels: A = 203, B = 295, C = 318, D = 352.

6 The notation X-X means that we compare two different sets, e.g., A and D.

7
*U* values were ranging from 264,300,345,444.0 to 6,185,043,831,303.0; *p* = 0.0 for all combinations, except for C-D (*p* = 2.176*e* − 154). Medians of the sets in attention strength: A = 8.228*e* − 22, B = 1.154*e* − 23, C = 1.0, D = 1.0.

8
https://spacy.io/.

9 The list of words used to filter noun phrases [‘right’, ‘left’, ‘top’, ‘bottom’, ‘the left’, ‘the right’, ‘the top’, ‘the bottom’, ‘the back’, ‘the front’, ‘back’, ‘front’, ‘far’, ‘the far’, ‘close’, ‘the close’].

10 We take the best performing layer (40) of the microsoft/deberta-xlarge-mnli model.

11 Note that some descriptions might be not necessarily global, e.g. “a cat that has whiskers and ears”. Thus, there is a noise in the model because there is no guarantee that descriptions only describe details at a scene level.

12
https://github.com/yahoo/object_relation_transformer
